# Identification of Anti-ErbB2 Dual Variable Domain Immunoglobulin (DVD-Ig™) Proteins with Unique Activities

**DOI:** 10.1371/journal.pone.0097292

**Published:** 2014-05-13

**Authors:** Jinming Gu, Jinsong Yang, Qing Chang, Xiaoqing Lu, Jieyi Wang, Mingjiu Chen, Tariq Ghayur, Jijie Gu

**Affiliations:** 1 AbbVie Bioresearch Center, R&D, Worcester, Massachusetts, United States of America; 2 Cancer Research, R&D, AbbVie Inc., North Chicago, Illinois, United States of America; Tulane University, United States of America

## Abstract

Inhibiting ErbB2 signaling with monoclonal antibodies (mAbs) or small molecules is an established therapeutic strategy in oncology. We have developed anti-ErbB2 Dual Variable Domain Immunoglobulin (DVD-Ig) proteins that capture the function of a combination of two anti-ErbB2 antibodies. In addition, some of the anti-ErbB2 DVD-Ig proteins gain the new functions of enhancing ErbB2 signaling and cell proliferation in N87 cells. We further found that two DVD-Ig proteins, DVD687 and DVD688, have two distinct mechanisms of actions in Calu-3 and N87 cells. DVD687 enhances cell cycle progression while DVD688 induces apoptosis in N87 cells. Using a half DVD687, we found that avidity may play a key role in the agonist activity of DVD687 in N87 cells.

## Introduction

ErbB2 is one of the four members of the ErbB family of receptor tyrosine kinases (RTKs). ErbB2 signaling plays a key role in development and in certain diseases, such as cancer [1]–[4]. For example, a significant portion of human breast, ovarian, and gastric cancer cells overexpress ErbB2 or have ErbB2 gene amplification [5]–[8]. Downstream of ErbB signaling, there are multiple pathways, including PI3K/AKT, Ras/MAPK, and MEK/Erk pathways, which control cell proliferation, growth, differentiation, and apoptosis [9].

The ErbB family members have multiple ligands, including epidermal growth factor (EGF), Heregulin, Betacellulin, and TGFα [10]–[12]. Upon ligand binding, they form homodimers and/or heterodimers, which induce receptor internalization and/or intracellular signaling [11], [13]. There is a significant amount of crosstalk among ErbB family members and other cell receptor tyrosine kinases, such as cMet and IGF1R, in cancer progression and drug resistance [14]–[19].

There have been extensive efforts to develop drugs that could specifically target ErbB2 signaling pathways over the last few decades [20]–[22]. Among them, the most successful are Trastuzumab [23], [24] and Lapatinib [25]. Trastuzumab is an anti-human ErbB2 monoclonal antibody (mAb) developed by Genentech that was approved by the FDA in 1998. Trastuzumab has shown significant efficacy in human cancer patients with ErbB2 overexpression [26]. Lapatinib is a small molecule developed by GlaxoSmithKline that targets both the ErbB2 and EGFR signaling pathways. Approved by the FDA in 2007, Lapatinib has been used to treat patients with advanced or metastatic breast cancer whose tumors overexpress ErbB2 [27].

More recently, Genentech has developed another anti-ErbB2 antibody, Pertuzumab, which targets domain II of the extra cellular domain (ECD) of ErbB2 and inhibits ErbB dimerization [28], [29]. Unlike Trastuzumab, which binds to domain IV of the ErbB2 ECD, Pertuzumab shows limited efficacy in human patients. However, when Trastuzumab and Pertuzumab were administered in combination, they showed significant synergies in both preclinical models and the clinic [30]–[32]. Because of this synergy, in June 2012 the FDA approved the Trastuzumab and Pertuzumab combination therapy for treating ErbB2-positive metastatic breast cancer.

After more than 25-years in development, bispecific antibodies have emerged as the next generation antibody-based therapeutics and have become intensively investigated preclinically. There are more than 50 recombinant bispecific antibody formats described in the literature [33]. A number of bispecific antibodies are currently in clinical studies, including MM111 (ErbB2/ErbB3) [34] and MEHD-7945A (EGFR/ErbB3) [35].

DVD-Ig technology is a bispecific platform for generating therapeutics having drug-like properties similar to those of mAbs that could be used to bind two different epitopes of the same target [36]–[40]. Various DVD-Ig molecules have shown efficacy in a number of preclinical models [39], [41], [42].

We have generated and characterized eight anti-ErbB2 DVD-Ig proteins that have the variable domains of two different anti-ErbB2 antibodies. Suprisingly, our data demonstrate that some of the DVD-Ig molecules retain the antagonist activities of both parental antibodies while others have strong agonist activities.

## Materials and Methods

### Construction, Expression and Purification of Anti-ErbB2 DVD-Ig Proteins, Anti-ErbB2/VEGF-A DVD-Ig Proteins, as well as Half DVD-Ig Proteins, Half-DVD687 and Half-DVD688

The anti-ErbB2 DVD-Ig proteins were generated as described previously [38], [39]. Briefly, the VH (GenBank: GM685464.1) and VL (GenBank: GM685466.1) sequences of a first anti-ErbB2 antibody (mAb1) and the VH (GenBank: HC359024.1) and VL (GenBank: HC359025.1) sequences of a second anti-ErbB2 antibody (mAb2) were linked with a short (ASTKGP) or a long (TVAAPSVFIFPP) linkers and expressed with a human IgG1 heavy chain or κ light chain constant domains. In table 1, LL indicates heavy chain long linker and light chain long linker; LS indicates heavy chain long linker and light chain short linker; SL indicates heavy chain short linker and light chain long linker; SS indicates heavy chain short linker and light chain short linker. The anti-ErbB2/anti-vascular endothelial growth factor A (VEGF-A) DVD-Ig proteins DVD37 and DVD38 were generated as described previously [38], [39]. Briefly, DVD37 and DVD38 were generated with the VH and VL sequences mAb1 and the VH (GenBank: HC869889.1) and VL (GenBank: HC869896.1) sequences of an anti-VEGF-A antibody (mAb3) with a short (ASTKGP) linker. For half-DVD687 and half-DVD688, the VH sequences of DVD687 or DVD688 were PCR cloned into a half DVD-Ig protein expression construct, which was built with two mutations (C220S and C226S) in the hinge region and four mutations in C_H_3 region (P395A, F405R, Y407R, and K409D) to disrupt the formation of the homodimer of two heavy chains [36], [43]. The plasmids encoding the HC of half DVD-Ig molecules and LC of DVD-Ig molecules were transiently expressed in human embryonic kidney 293 cells (American Type Culture Collection (ATCC), Manassas, VA) and purified using protein A chromatography (Pierce, IL) according to manufacturer's instructions. The resulting half-DVD-Ig proteins contain only a single arm of a full-length antibody as confirmed by size exclusion chromatography (SEC). All DVD-Ig proteins (except DVD689, DVD691, and DVD692) and half-DVD-Ig proteins were confirmed to be <10% aggregates by SEC. In all the assays, monoclonal antibodies were compared at the same molarity as in combination, e.g., 10 nM mAb1 was compared to 10 nM mAb2, 10 nM mAb1+10 nM mAb2, or 10 nM DVD-Ig proteins.

**Table 1:** Generation of anti-ErbB2 DVD-Ig molecules.

ID	Outer domain	Inner domain	Linker	Titer (mg/L)	SEC % of monomer
DVD687	mAb1	mAb2	SS	110	90
DVD688	mAb2	mAb1	SS	89	91
DVD689	mAb1	mAb2	LL	93	86
DVD690	mAb2	mAb1	LL	66	92
DVD691	mAb1	mAb2	LS	101	86
DVD692	mAb2	mAb1	LS	83	85
DVD693	mAb1	mAb2	SL	94	92
DVD694	mAb2	mAb1	SL	82	93

### Cell Lines and Cell Culture Conditions

Calu-3, N87 and MDA-MB175 cells were obtained from the American Tissue Culture Collection (ATCC, VA). All cells were maintained in DMEM medium supplemented with 10% fetal bovine serum (FBS), 50 units/mL penicillin, and 50 µg/mL streptomycin.

293G cells stably transfected with both YFP and luciferase-tagged ErbB2 (C-terminus deletion) were maintained in DMEM medium supplemented with 10% fetal bovine serum (FBS), 50 units/mL penicillin, 50 µg/mL streptomycin, 500 µg/ml G418, and 250 µg/ml hygromycin.

### Binding Analysis

For ELISA, plates were coated with 1 µg/ml of anti-his antibody (Invitrogen, CA) in carbonate buffer at 4°C overnight. After blocking with Superblock (Pierce, CA) at room temperature for an hour, His-tagged ErbB2 proteins (R&D Systems, MN) were added in 1% BSA in PBS at room temperature. After washing, various concentrations of antibodies were added for 1 hour at room temperature and captured antibodies were detected by HRP-conjugated goat-anti-human antibodies (Jackson Immunoresearch, PA).

For cell based binding studies, cells were harvested with cell dissociation buffer (Invitrogen, CA) and incubated with different concentrations of antibodies or DVD-Ig proteins on ice for 45 minutes in PBS. Cells were washed and incubated with PE-conjugated goat-anti-human antibodies (Jackson Immunoresearch, PA) on ice for 30 minutes. For competition binding, FITC-labeled antibodies were pre-incubated with non-labeled antibodies on ice for 15 minutes. Cells were then incubated with this antibody mixture on ice for 45 minutes. Cells were washed and analyzed on a FACS Calibur with FlowJo. Median fluorescence intensity (MFI) was used for analysis.

For surface plasmon resonance (SPR) analysis, Biacore instruments with Biacore software (GE Healthcare Life Sciences, Piscataway, NJ) were used to run the kinetic and stoichiometry experiments. Goat antibodies specific to the Fc region of hu IgG were used for mAb or DVD-Ig protein capture. Fc specific antibodies were covalently immobilized on the carboxy methyl dextran matrix of the CM5 biosensor chip via amino groups using an amine coupling kit from GE Healthcare Life Sciences according to the manufacturer's protocol. Approximately 5000 RU of goat anti-human IgG Fc mAbs was immobilized on the chip surface on flow cells 2, 3 and 4. A modified CM surface with similarly conjugated goat IgG antibodies in flow cell 1 was used as a reference surface. Antibodies or DVD-Ig proteins were diluted in the running buffer, HBS-EP+0.1 mg/mL BSA (HBS was diluted from HBS-EP+10x; GE Healthcare Life Sciences, NJ), and were injected over the goat anti-human IgG Fc surface on flow cells to achieve capture level of ∼50–200 RU. The antigen binding kinetic was tested at the following recombinant ErbB2 (R&D Systems, MN) concentrations: 200, 100, 50, 25, 12.5, 6.75, 3.125, 1.56, 0.78 and 0 nM. Association rate was evaluated for 5 min and dissociation phase consisted of continued flow of HBS-EP+, 0.1 mg/mL BSA buffer, at 50 µL/min for10 min. Immobilized surfaces were regenerated with two 30 s pulses of 10 mM glycine, pH 1.5 before injection of the next sample. The reference surface response was subtracted from the reaction surface data in order to eliminate change in the refractive index and injection noise. Association and dissociation rate constants, as well as overall K_D_ were calculated by the instrument evaluation software based on the values extracted from the data using global 1∶1 fit analysis (allowing identical values for each parameter in the data set, except for R_max_ that was set local due to variation in capture level). The goodness-of-fit between the 1∶1 model fitted curve and the experimental data was expressed by the evaluation software as chi^2^.

### Immunoblot Analysis

For cell signaling studies, cells were plated into 6-well plates and incubated overnight. Cells were serum-starved for 24 hours and pre-incubated with 100 nM antibodies or DVD-Ig proteins each for 2 hours and stimulated with 10 nM HRG for 10 minutes and then harvested. Cells were lysed with RIPA buffer (Sigma-Aldrich, MO) supplemented with protease and phosphatase inhibitor cocktails (Roche Diagnostics, IN). Cell lysate proteins were resolved by SDS-PAGE and immunoblots were probed with antibodies against phosphorylated Tyrosine (BD Biosciences, CA), EGFR (NeoMarker, Thermo Fisher, CA), ErbB3, phosphorylated extracellular signal-regulated kinase (pERK) 1/2 (Thr^202^/Thr^204^), ERK1/2, AKT (Santa Cruz, CA), ErbB2, phosphorylated AKT (pAKT; Ser^473^), followed by incubation with IRDye 700 conjugated goal anti-mouse and IRDye 800 conjugated goat anti-rabbit (LI-COR, NE). Total protein was detected with anti-PCNA (Santa Cruz, CA) followed by IRDye680 CW conjugated goat anti-mouse (LI-COR, NE). Blots were visualized using an Odyssey Imaging system (LI-COR, NE).

For ErbB receptor profiling studies, log-phase cells were cultured in 6-well plates and were washed with ice-cold PBS quickly and lysed in the lysis buffer containing 50 mM Tris (pH7.5), 1 mM EDTA (pH 8.0), 1% Triton X-100, 150 mM NaCl, a protease inhibitor cocktail (Roche Diagnostics, IN) and phosphatase inhibitor (Roche Diagnostics, IN) followed by the same SDS-PAGE and immunoblot procedure.

### Co-immunoprecipitation Assay

Calu-3 or N87 cells were serum-starved for 24 hours and pre-incubated with 30 nM antibodies and DVD-Ig proteins each for 2 hours and stimulated with 10 nM HRG for 10 minutes and then harvested. Cells were lysed with RIPA buffer (Sigma-Aldrich, MO) supplemented with protease and phosphatase inhibitor cocktails (Roche Diagnostics, IN). The cell lysate were incubated with anti-ErbB2 conjugated Protein A/G beads (Santa Cruz, CA) in the cold room for 90 minutes. The beads were then washed three times with RIPA buffer and boiled in 2X SDS loading buffer (Invitrogen, CA) for five minutes. Proteins were resolved by SDS-PAGE and immunoblots were probed with antibodies against EGFR (NeoMarker, Thermo Fisher, CA), ErbB2 (Cell Signaling, MA), ErbB3 (Santa Cruz, CA), and PCNA (Santa Cruz, CA).

293G cells stably transfected with both YFP and Luciferase tagged ErbB2 (C-terminus deletion) were treated with 30 nM DVD-Ig protein or mAbs for two hours and then harvested. Cells were lysed with RIPA buffer (Sigma-Aldrich, MO) supplemented with protease and phosphatase inhibitor cocktails (Roche Diagnostics, IN). The cell lysate were incubated with anti-YFP conjugated Protein A/G beads (Santa Cruz, CA) in the cold room for 90 minutes. The beads were then washed three times with RIPA buffer and boiled in 2X SDS loading buffer (Invitrogen, CA) for five minutes. Proteins were resolved by SDS-PAGE and immunoblots were probed with antibodies against YFP (Santa Cruz, CA), Luciferase (Thermo Fisher, CA), and PCNA (Santa Cruz, CA).

### Cell Proliferation Assays

For ^3^H thymidine incorporation assays, cells cultured in 96-well tissue-culture plates (1000∼5000 cells per well) in medium were treated with serial diluted antibodies or DVD-Ig proteins for 96 hours, then ^3^H thymidine (1 µCi per well) was added and incubated overnight. The cells were harvested and the amount of ^3^H thymidine uptake was measured using microplate scintillation and a luminescence counter.

For colony formation assay, N87 cells (1,000/well) were seeded into 96-well plates and incubated overnight. Antibodies were added at the doses indicated and cells were treated for ten days. Following washing, cells were fixed with 4% formaldehyde, and stained with 0.1% crystal violet. Stained crystal violet was extracted with 10% acetic acid and quantitated at A540.

### Apoptosis Assay

Calu-3 or N87 cells (10,000/well) were seeded into 6-well plates and incubated overnight. Antibodies or DVD-Ig proteins were added at 100 nM and apoptotic cells were counted for the subsequent three days by FITC-Annexin V (BD Biosciences, CA) and propidium iodide (Sigma-Aldrich, MO) labeling according to manufacturer’s protocol.

### BrdU-incorporation Assay

Calu-3 or N87 cells (100,000/well) were seeded into 6-well plates and incubated overnight. Antibodies or DVD-Ig proteins were added at 100 nM and cells were treated for two days. Cells were pulse-labeled with 10 mM BrdU (BD Biosciences, CA) for 30 minutes at 37°C and then harvested and stained with BrdU-FITC (BD Biosciences, CA) according to manufacturer’s protocol.

## Results

### Generation of Anti-ErbB2 DVD-Ig Proteins

To test whether we could capture the synergistic effect of two anti-ErbB2 antibodies via the DVD-Ig platform, we used the variable domains of two different anti-ErbB2 antibodies to generate eight DVD-Ig proteins differing in the orientation of the two variable domains and linkers used (see Materials and Methods for details) (Table 1). We then transiently expressed these DVD-Ig proteins in 293 cells and purified them using Protein A columns. We found all eight DVD-Ig molecules show high expression levels and low aggregation (Table 1), making them suitable for further characterization.

### ErbB2 Binding Characterization of Anti-ErbB2 DVD-Ig Proteins

We then tested the binding of these DVD-Ig proteins to ErbB2. For ELISA binding, plates were coated with ErbB2-ECD to capture these DVD-Ig proteins and the single antibodies. The results show that all eight DVD-Ig proteins have similar binding affinity to ErbB2-ECD as compared to single antibodies (Table 2). The EC50 of the DVD-Ig proteins ranged from 0.16 to 5.96 nM while the EC50 for the two single antibodies and the combination were 0.68, 3.84, and 0.93 nM respectively. For FACS binding, DVD-Ig proteins and single antibodies were tested for their binding to N87 cells, which overexpress ErbB2. The results indicated that most of the DVD-Ig proteins have binding affinity similar to that of the single antibodies while DVD691 and DVD693 show more than 10 fold increase in binding compared to the single antibodies (Table 2).

**Table 2:** Characterization of ErbB2 binding of anti-ErbB2 DVD-Ig molecules.

Unique ID	ELISA Binding		FACS Binding		Blocking capacity (%)			
	B_MAX_	EC50 (nM)	B_MAX_	EC50 (nM)	ELISA		FACS	
	(Absorbance)		(MFI)		Biotin-1	Biotin-2	Biotin-1	Biotin-2
mAb1	*2.79*	*0.68*	830	1.6	*90*	*−34*	*96*	*−36*
mAb2	*2.49*	*3.84*	938	1.51	*−1*	*81*	*−6*	*97*
mAb1+mAb2	*2.83*	*0.93*	1140	2.57	*90*	*75*	*N/A*	*N/A*
DVD687 (1/2 SS)	3	1.71	857	4.79	*90*	*50*	*N/A*	*N/A*
DVD688 (2/1 SS)	3.13	3.59	1085	4.03	*14*	*91*	*N/A*	*N/A*
DVD689 (1/2 LL)	2.96	0.6	958	1.69	*90*	*91*	*N/A*	*N/A*
DVD690 (2/1 LL)	3.15	0.48	N/A	N/A	*68*	*91*	*N/A*	*N/A*
DVD691 (1/2 LS)	3.23	0.19	805	0.09	*90*	*88*	*N/A*	*N/A*
DVD692 (2/1 LS)	3.17	5.96	1083	2.94	*15*	*66*	*N/A*	*N/A*
DVD693 (1/2 SL)	3.22	0.16	800	0.15	90	90	96	96
DVD694 (2/1 SL)	3.39	0.53	1204	2.39	88	91	89	97

Since both variable domains of the DVD-Ig protein bind to ErbB2, we next determined whether both variable domains retain their activities in the DVD-Ig format. To test this, we utilized a competition-binding assay, where Biotin-labeled mAb1 or mAb2 was used to compete with unlabeled DVD-Ig proteins or single antibodies. As predicted, Biotin-labeled mAb1 or mAb2 could strongly compete with unlabeled mAb1 or mAb2 respectively (Table 2). Biotin-labeled mAb1 or mAb2 show variable ability to block the binding of DVD-Ig protein binding to ErbB2-ECD (Table 2). For example, Biotin-labeled mAb1 could block 90% of binding of DVD687 to ErbB2-ECD while Biotin-labeled mAb2 could only block 50% of DVD687 binding (Table 2), suggesting that both variable domains are active but most of the ErbB2 binding affinity comes from mAb1. Overall, the competition assay results suggest that both variable domains are active in these DVD-Ig proteins. We further tested mAbs’ and DVD-Ig proteins’ binding affinity to ErbB2 via Biacore analysis. The results show that the DVD-Ig proteins retain the binding affinity of the parental mAbs (Table 3).

**Table 3:** Biacore analysis of ErbB2 binding affinity of anti-ErbB2 DVD-Ig molecules.

Unique ID	Biacore Kinetic Rate Parameters		
	On-rate (M-1s-1)	Off-rate (s-1)	K_D_ (nM)
mAb1	1.09E+05	2.01E-04	1.84
mAb2	7.03E+04	4.21E-04	5.99
DVD687 (1/2 SS)	1.03E+05	7.80E-05	0.76
DVD688 (2/1 SS)	4.85E+04	2.66E-04	5.49
DVD689 (1/2 LL)	N/A	N/A	N/A
DVD690 (2/1 LL)	N/A	N/A	N/A
DVD691 (1/2 LS)	1.18E+05	6.10E-05	0.51
DVD692 (2/1 LS)	4.66E+05	1.35E-04	2.9
DVD693 (1/2 SL)	1.26E+05	1.86E-05	0.15
DVD694 (2/1 SL)	7.89E+04	1.46E-04	1.84

### Anti-ErbB2 DVD-Ig Molecules with Different Linkers and Variable Domain Orientations Show Different Effects on Cell Proliferation

**Figure 1:**
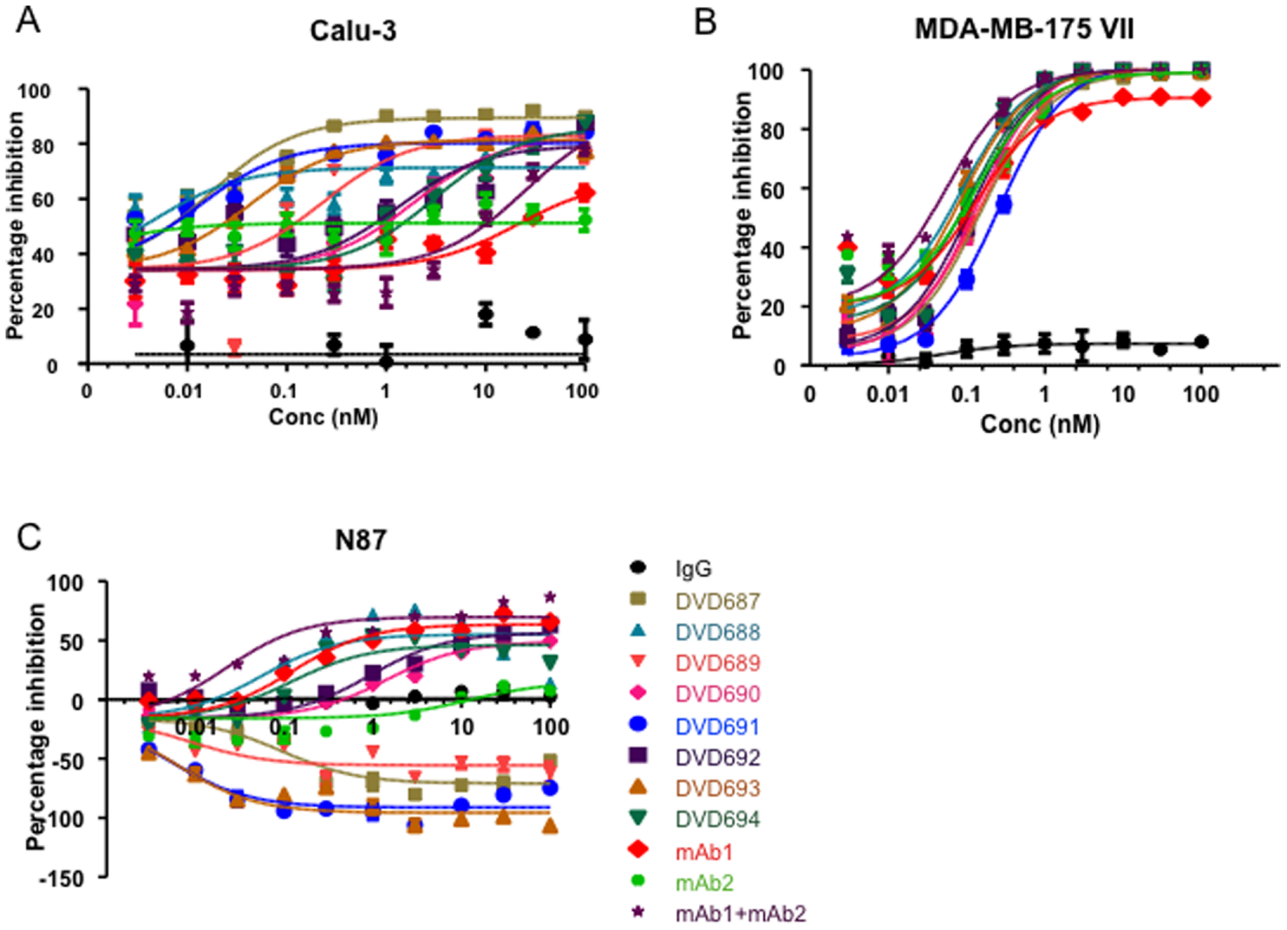
Anti-ErbB2 DVD-Ig proteins inhibit cell proliferation. (A) Calu-3, (B) MDA-MB-175 VII, and (C) N87 cells were treated with indicated dosages of DVD-Ig proteins, mAbs, or combination for 96 hrs. Cell proliferation was measured with ^3^H thymidine incorporation assays as described in Materials and Methods. Three independent experiments with triplicates were performed. One representative experiment is shown here. The error bars indicate standard deviation from the mean.

**Figure 2:**
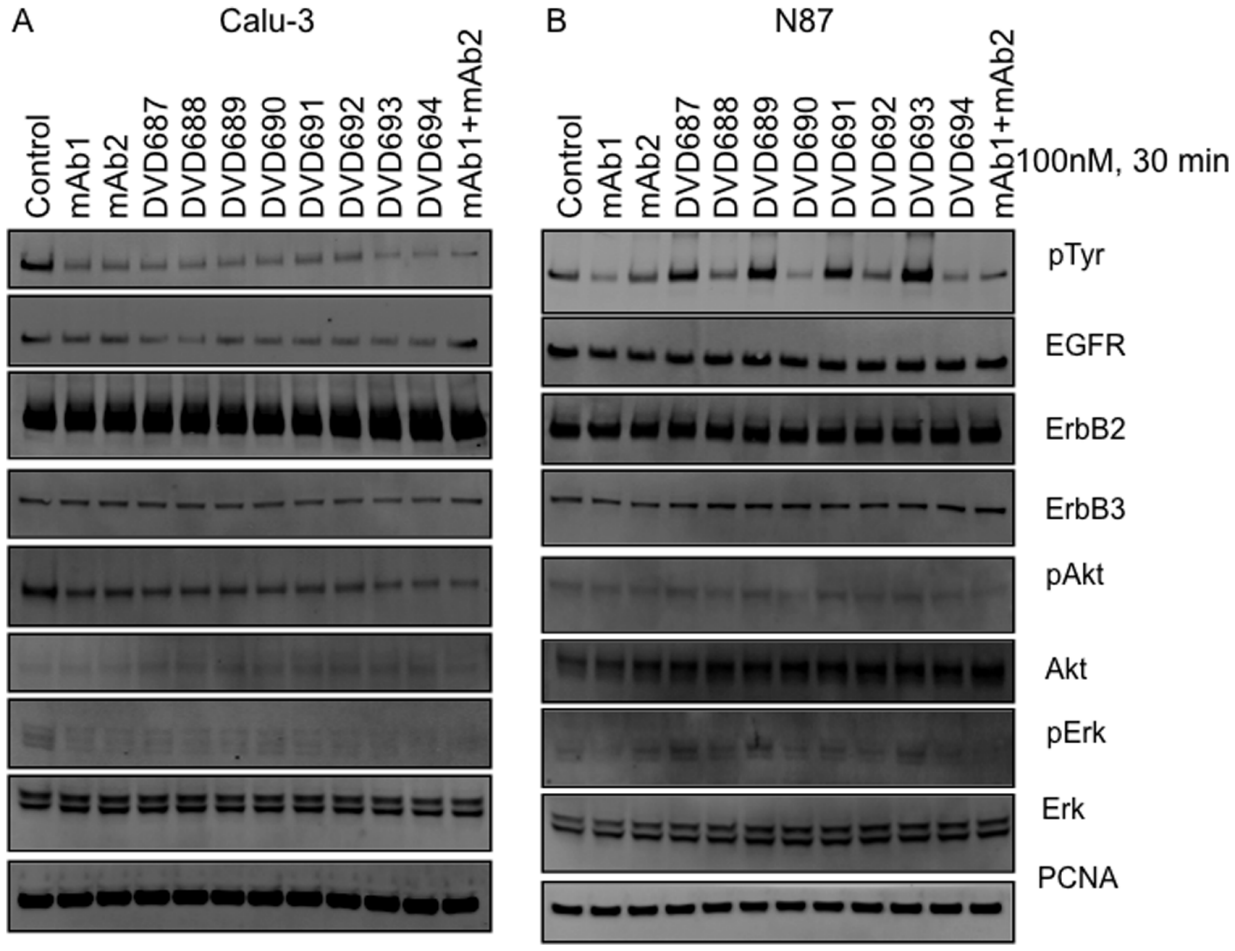
Anti-ErbB2 DVD-Ig proteins in cell signaling assay. (A) Calu-3 and (B) N87 cells were cultured in medium containing 1% FBS were treated with 100nM antibodies or DVD-Ig proteins for 30 minutes. Cells were lysed, proteins were separated on SDS-PAGE gels and analyzed via western blot. Three independent experiments were performed. One representative experiment is shown here.

To test whether the anti-ErbB2 DVD-Ig proteins retained the cell proliferation inhibition activity of mAb1 and mAb2, we screened the eight DVD-Ig proteins in a panel of human cancer cell lines via cell proliferation assay. Among the cells that responded to mAb1 and mAb2, we found three patterns of anti-ErbB2 DVD-Ig response: (1) in Calu-3 cells, eight DVD-Ig proteins show variable levels of cell proliferation inhibition with several of the DVD-Ig proteins having significantly improved IC50 compared to the single antibodies and the antibodies in combination (Fig. 1A); (2) in MDA-MB175 VII cells, all eight DVD-Ig proteins show similar IC50 as the single antibodies and the antibodies in combination (Fig. 1B); and (3) in N87 cells, four DVD-Ig proteins with mAb2 variable domains on the outside show cell proliferation inhibition, with DVD688 inhibition being similar to that of mAb1 and the antibodies in combination while the other four DVD-Ig proteins with mAb1 variable domains on the outside show enhanced cell proliferation as compared to that of mAb1 and the antibodies in combination (Fig. 1C).

### Anti-ErbB2 DVD-Ig Proteins with Different Linkers and Variable Domain Orientations Show Different Effects on ErbB2 Signaling

Since we have seen that some anti-ErbB2 DVD-Ig proteins are antagonists while others are agonists in N87 cells, we next tested these DVD-Ig proteins in a cell signaling assay. Calu-3 and N87 cells cultured in medium containing 1% FBS were treated with various DVD-Ig proteins and single antibodies for 30 minutes. In Calu-3 cells, single antibodies and DVD-Ig proteins could inhibit pTyr, pAkt, and pErk signaling (Fig. 2A). Interestingly, those four DVD-Ig proteins that showed enhanced cell proliferation significantly promoted pTyr, pAkt, and pErk signaling (Fig. 2B), suggesting that they enhance N87 cell proliferation through an ErbB2 signaling pathway. Together, the signaling data are consistent with the cell proliferation data.

### DVD687 and DVD688 have Different Effects on ErbB Heterodimer and Homodimer Formation

**Figure 3:**
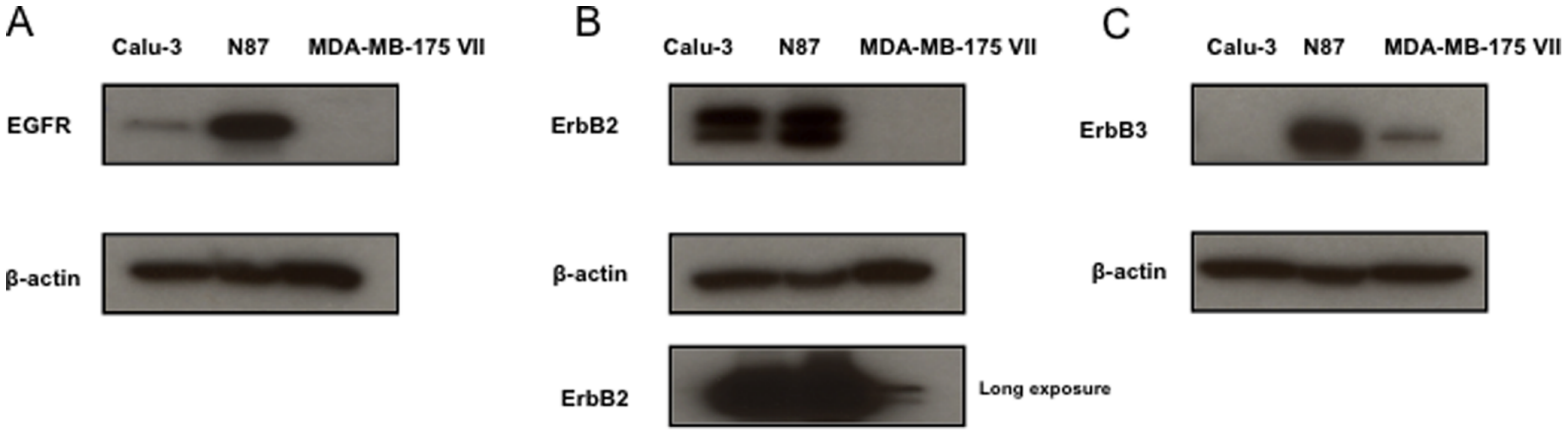
ErbB receptor expression. (A) EGFR, (B) ErbB2, and (C) ErbB3 protein expression was analyzed in Calu-3, N87, and MDA-MB175 VII cells. Log-phase proliferating subconfluent cells were lysed, proteins were separated on SDS-PAGE gels and analyzed via western blot. Three independent experiments were performed. One representative experiment is shown here.

Since the three different cell lines show different responses to anti-ErbB2 DVD-Ig proteins, we profiled the ErbB family proteins in these three cell lines. Interesting, the results show that (1) N87 cells express EGFR at a much higher level compared to Calu-3 and MDA-MB175 VII cells (Fig. 3A); (2) both Calu-3 and N87 cells express ErbB2 at a much higher level compared to MDA-MB175 VII cells (Fig. 3B); and (3) N87 cells express ErbB3 at a much higher level compared to Calu-3 and MDA-MB175 VII cells (Fig. 3C).

**Figure 4:**
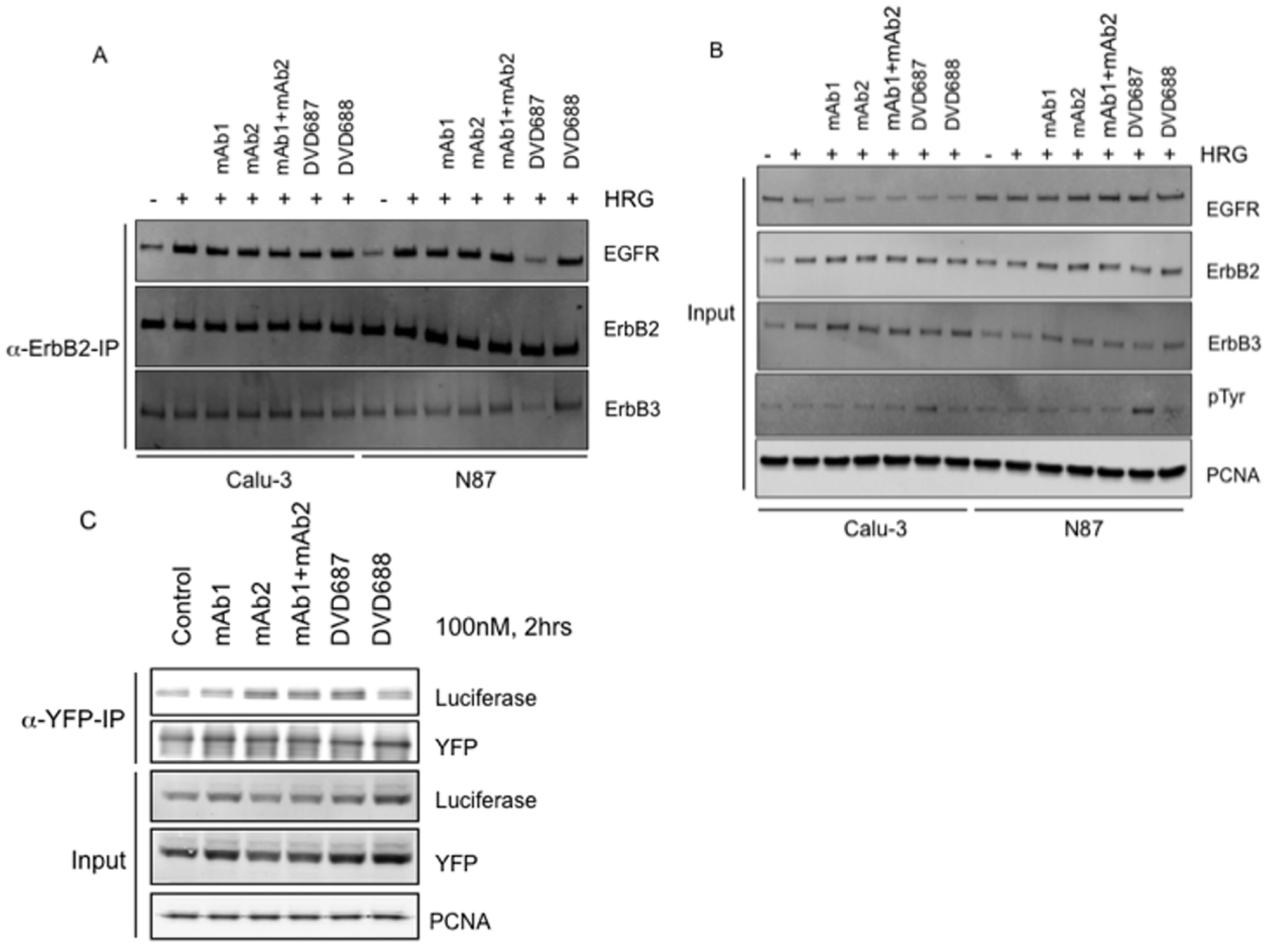
DVD687 affects EGFR/ErbB2 and ErbB2/ErbB3 heterodimer and ErbB2 homodimer formation. (A) Calu-3 and N87 cells were serum starved, treated with DVD-Ig proteins or single antibodies for two hours, and then stimulated with heregulin for ten minutes. Cells were lysed and endogenous ErbB2 protein complex was immunoprecipitated with an anti-ErbB2 (C-terminus) antibody. Endogenous EGFR and ErbB3 co-immunoprecipitated with ErbB2 were detected via western blot. (B) Pre-co-immunoprecipitation cell lysate was used as an input. (C) YFP- and luciferase-tagged ErbB2 overexpressed 293 cells were treated with DVD-Ig proteins or single antibodies for two hours and then ErbB2 complex was immunoprecipitated with anti-YFP antibody. Immunoprecipitated YFP-tagged ErbB2 (c-terminus deletion) and co-immunoprecipitated luciferase-tagged ErbB2 (c-terminus deletion) were detected via western blot. Three independent experiments were performed. One representative experiment is shown here.

We next asked whether DVD687 and DVD688, which have different variable domain orientations, would have different effects on ErbB2/ErbB3 and/or EGFR/ErbB2 heterodimer formation. Calu-3 and N87 cells were serum starved, treated with DVD-Ig proteins or single antibodies for two hours, and then stimulated with heregulin for ten minutes. Endogenous ErbB2 protein complex was immunoprecipitated with an anti-ErbB2 (C-terminus) antibody. Interestingly, the results show that in N87 cells DVD687 has less EGFR pulled down than DVD688 and less ErbB3 pulled down as well (Fig. 4A), suggesting that DVD687 could specifically disrupt both EGFR/ErbB2 and ErbB2/ErbB3 heterodimer formation. This phenomenon was not observed in Calu-3 cells (Fig. 4A). Using a cell lysate input western blot, we found that DVD687 could strongly enhance phospho-tyrosine signaling in N87 cells (Fig. 4B), which is consistent with the results illustrated in Fig. 2.

To test the effect of anti-ErbB2 DVD-Ig proteins’ effect on ErbB2 homodimer formation, we generated a 293 stable cell line that expresses both YFP- and luciferase-tagged ErbB2 (C-terminus deletion). The cells were treated with DVD-Ig proteins or single antibodies for two hours and then ErbB2 complex was immunoprecipitated with anti-YFP antibody. The results indicate that DVD687 could induce ErbB2 homodimer formation as compared to DVD688 (Fig. 4C), and comparable to mAb2 and the combination of mAb1 and mAb2. Taken together, the results shown in Fig. 4A and 4C suggest that unlike Calu-3 and MDA-MB175 VII cells, N87 cells have a high level of EGFR/ErbB2 and ErbB2/ErbB3 heterodimer formation due to their high level of expression of their three receptors. DVD687 could specifically disrupt the EGFR/ErbB2 and ErbB2/ErbB3 heterodimer formation, thus inducing more ErbB2 homodimer formation, which may (1) enhance ErbB2 signaling and then (2) then promote cell proliferation.

### DVD687 and DVD688 Show Different Mechanism of Actions (MOAs)

**Figure 5:**
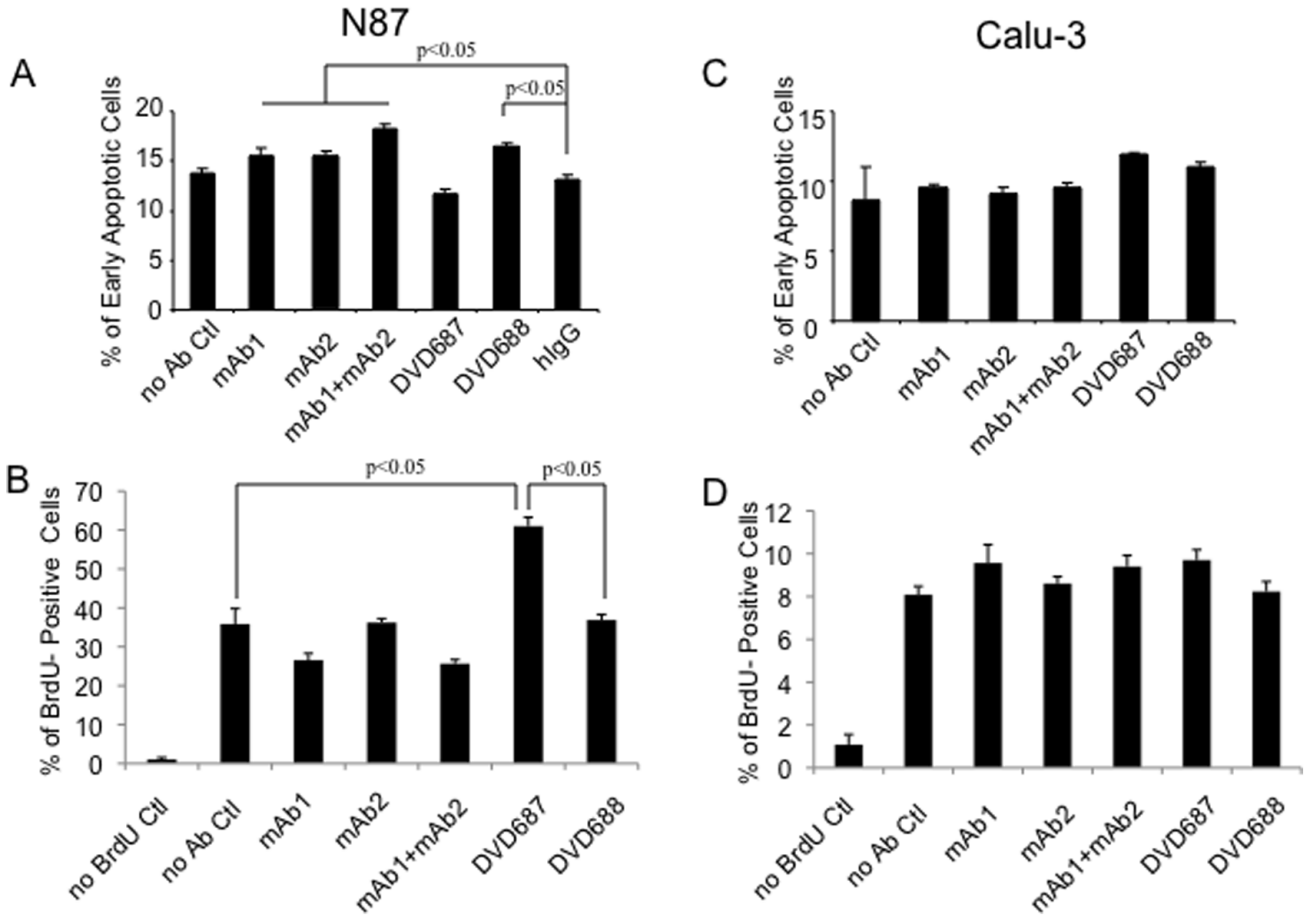
DVD687 and DVD688 show different mechanism of actions (MOAs). Apoptosis assay was used to analyze (A) N87 or (C) Calu-3 cells after DVD-Ig proteins, mAbs, or combination treatment. BrdU-incorporation assay was used to analyze (B) N87 or (D) Calu-3 cells after DVD-Ig proteins, mAbs, or combination treatment. Three independent experiments with triplicates were performed. One representative experiment is shown here. The error bars indicate standard deviation from the mean. p value was calculated via student T-test.

To determine the question of why DVD687 and DVD688 have different effects on N87 cell proliferation, we performed apoptosis and cell cycle analysis. Interestingly, the results show that a 24-hour DVD688 treatment significantly induced apoptosis while a similar treatment with DVD687 had no effect (Fig. 5A). In the cell cycle assay, DVD687 showed a significant increase in BrdU-positive cells while DVD688 had little effect (Fig. 5B). Together, these data are consistent with what observed in the cell proliferation assay: DVD687 induces cell cycle progression but has no effect on apoptosis which resulted in the enhancement of N87 cell proliferation; DVD688 induces apoptosis but has little effect on cell cycle, which resulted in the inhibition of N87 cell proliferation. In contrast, DVD687 and DVD688 show similar effects in Calu-3 cells with regard to both apoptosis and cell cycle progression (Fig. 5C and 5D), which explains their similar effects in inhibiting the Calu-3 cell proliferation.

### Half DVD687 Loses Agonist Effect

**Figure 6:**
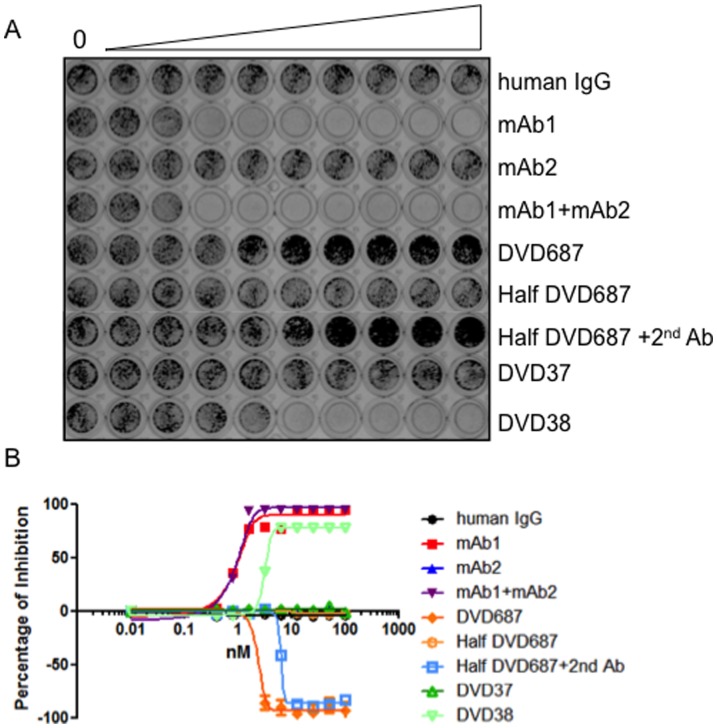
Half DVD687 loses agonist effect. N87 cells were treated with indicated dosages of antibodies, DVD687, half DVD687, DVD37, or DVD38. After 10 days, cells were stained with crystal violet (A) and then quantitated (B). Three independent experiments with triplicates were performed. One representative experiment is shown here. The error bars indicate standard deviation from the mean.

To determine the question of whether avidity is required for DVD687’s agonist activity, we generated and tested a half DVD687. We then tested the half DVD687 together with the whole DVD687 in the N87 cell proliferation assay. Surprisingly, the half DVD687 had completely lost the agonist activity of the whole DVD687 (Fig. 6A&B). However, when goat-anti-human antibody was added together with half DVD687, thereby simulating an intact DVD687, the half DVD-Ig proteins joined together regained the agonist effect (Fig. 6A and 6B). We then determined whether the pairing of mAb1with an unrelated mAb could create a similar agonist. mAb1 was paired with a control mAb3 to generate DVD37 and DVD38. DVD38 (having a mAb1 variable domain as the outer domain) had N87 cell proliferation inhibition activity while DVD37 (having a mAb3 variable domain as the outer domain) had little N87 cell proliferation inhibition activity. mAb3 alone had no effect on N87 cell proliferation. Together, these data suggest that avidity, together with domain orientation, may be important for the functional agonist activity of DVD687 and that DVD687 needs both mAb1 and mAb2 functional to be an agonist.

## Discussion

We have generated anti-ErbB2 DVD-Ig proteins, some of which have the same functional activity as the parental mAb1 and mAb2 combination treatment and some of which possess new functions. For example, we identified four anti-ErbB2 DVD-Ig proteins that functions as agonists in N87 cells. This proves that DVD-Ig proteins provide a platform that could generate bispecific molecules in an efficient way by screening combinations of single antibodies and incorporating the variable domains into a DVD-Ig framework. This was highly unexpected because the parental antibodies from which the two variable domains were derived function are antagonists as single antibodies. Recently, a study from Genentech showed that one can generate agonist activity out of Trastuzumab, which is normally a strong antagonist, when its Fab regions are crosslinked [44].

Trastuzumab and Pertuzumab bind to the different domains of ErbB2 [45], [46]. In addition, Trastuzumab and Pertuzumab inhibit cell proliferation and tumor growth with different mechanisms [14], [24], [29], [32], [47]. Interestingly, when incorporating Trastuzumab and Pertuzumab into DVD-Ig proteins, we observed a novel (agonist) function that is not achieved by simply combining Trastuzumab and Pertuzumab. We hypothesized that some of our DVD-Ig proteins could distort the conformation of the ErbB2 after binding and thus affect the homo/hetero-dimer formation and intracellular signaling. A co-crystallization of DVD-Ig protein and ErbB2 ECD may solve this mystery.

Our results indicated that linkers and orientations play important roles in DVD-Ig protein function. For example, the outer variable domains of a DVD-Ig protein generally bind to the antigen at a higher affinity than the inner variable domains. Significant differences were observed between DVD-Ig proteins with different variable domain orientations and linker lengths not only with regard to binding but also with regard to ErbB2 signaling and the inhibition of cell proliferation. For example, all four anti-ErbB2 DVD-Ig proteins with the mAb1 variable domains on the outside possessed strong agonist activity in N87 cells while the other four anti-ErbB2 DVD-Ig proteins were antagonists. In Calu-3 and MDA-MB175 VII cells, all eight anti-ErbB2 DVD-Ig proteins possessed different patterns with regard to the inhibition of cell proliferation.

One question remains: why do anti-ErbB2 DVD-Ig proteins show different patterns in N87 cells? Our co-immunoprecipitation assay results suggest that DVD687 prefers ErbB2 homodimer formation to EGFR-ErbB2 and ErbB2-ErbB3 heterodimer formation in N87 cells. However, this may not be the case in Calu-3 cells, which may be due to the difference in the expression of EGFR and ErbB3 on the cell surface. Our results further indicate that DVD687 and DVD688 have two distinctive mechanisms of action in N87 cells. DVD687 enhances cell cycle progression while DVD688 induces apoptosis. We hypothesize that when binding to the ErbB2 receptor, DVD687 and DVD688 may induce different ErbB2 conformational changes, due to the differences in their variable domain orientations, such that DVD687 behaves as an agonist while DVD688 behaves as an antagonist. Our data further demonstrates that the DVD-Ig protein avidity and its domain orientation may play key roles in the agonist activity of DVD687. Together, these data suggest that DVD687 may form a large complex with cell surface ErbB2 and enhance the ErbB2 homodimer formation, thereby inducing cell signaling and proliferation.

In summary, we report that anti-ErbB2 DVD-Ig proteins could inhibit cell proliferation similar to, if not better than, mAb1, mAb2, or the combination thereof. In addition, we identified several anti-ErbB2 DVD-Ig proteins that have unexpected agonist activity in N87 cells. Together, these results suggest that one can use DVD-Ig proteins not only to capture the functions of the two single antibodies in combination but also to obtain some new functions.
